# Effects of empowerment education on patients after percutaneous coronary intervention: A meta-analysis and systematic review

**DOI:** 10.1097/MD.0000000000033992

**Published:** 2023-06-09

**Authors:** Linbin Guo, Wanpeng Gao, Tianlin Wang, Xinjue Shan

**Affiliations:** a Graduate School, Tianjin University of Traditional Chinese Medicine, Tianjin, China; b Department of Emergency, The Second Affiliated Hospital of Tianjin University of Traditional Chinese Medicine, Tianjin, China; c First Teaching Hospital of Tianjin University of Traditional Chinese Medicine, National Clinical Research Center for Chinese Medicine Acupuncture and Moxibustion, Tianjin, China.

**Keywords:** empowerment education, meta-analysis, PCI, quality of life, systematic review

## Abstract

Empowerment education is a new nursing education model with great significance in the process of chronic disease rehabilitation, and a number of studies have found that it has good benefits for patients after percutaneous coronary intervention (PCI). But there is no meta-analysis on how empowerment education influence the life of patients after PCI.

**Aims:**

Our study intends to evaluate the impact of empowerment education on the quality of life, cognitive level, anxiety and depression level of patients after PCI.

**Design:**

Systematic review and meta-analysis, following PRISMA guidelines.

**Methods:**

RevMan5.4 software and R software were used for statistical analysis. Mean difference or standard mean difference was used as effect analysis statistic for continuous variables with 95% confidence intervals.

**Results:**

Six studies met the inclusion criteria, including 641 patients. The Exercise of Self-Care Agency Scale score of the experimental group was higher than that of the control group, with statistically significant difference. Empowerment education could increase the knowledge of coronary heart disease in patients after PCI, but the difference was not statistically significant.

**Conclusion:**

Significant effects of empowerment have been found in improving patients’ quality of life and self-care ability. Empowerment education could be a safe exercise option in PCI rehabilitation. However, the effect of empowerment on cognitive level for coronary heart disease and the depression needs to carry out more large-sample, multi-center clinical trials.

**Patient or public contribution:**

A data-analysis researcher and 3 clinicians are responsible for the writing, and no patients participated in the writing of this paper.

## 1. Introduction

Epidemiological data showed that cardiovascular disease currently had accounted for about one-third of all deaths worldwide. Although mortality rates for coronary heart disease (CHD) have declined in recent decades, CHD remains the leading cause of death in the United States and poses a significant economic burden.^[1]^ In all the treatments of CHD, percutaneous coronary intervention (PCI) is not only safe but also effective.^[2]^ However, PCI is only a mechanical recanalization of vessels, which cannot fundamentally correct the risk factors (such as smoking, hypertension, hyperlipidemia, etc.).^[3,4]^ In addition, many patients after PCI still suffer from chest tightness, fatigue and other uncomfortable symptoms due to various factors, such as the lack of knowledge of CHD, fear of the operation, anxiety and depression,^[5]^ which bring huge troubles to their lives.^[6]^ Therefore, it is important for patients after PCI to accept effective nursing education mode for postoperative rehabilitation.

WHO defines “empowerment” as individuals having access to information and resources related to their health, participating in decision-making and implementation, and working to change the factors that affect their health.^[7]^ This theory emphasizes “patient-centered.” Educators and patients are in a cooperative relationship, believing that patients are responsible for their own health conditions, while educators are only guides and information providers.^[8,9]^ Currently, studies on empowerment education have been widely carried out in patients with chronic diseases such as type 2 diabetes, cancer, and long-term dialysis for kidney disease. A number of meta-analyses and randomized controlled trials (RCTs) have confirmed its safety and effectiveness in the field of chronic disease rehabilitation.^[10–13]^ In the cardiovascular field, studies have shown that health programs based on empowerment theory can improve disease cognition and physical condition in the CHD patients.^[14,15]^ In recent years, due to the extensive development of PCI technology, more and more researchers began to try to apply empowerment education as a nursing mode to PCI postoperative education, in order to increase patients’ understanding of the disease, improve patients’ quality of life, reduce and prevent the occurrence of complications.^[16–21]^ But there is no meta-analysis on how empowerment education influence the life of patients after PCI. Therefore, we conducted a systematic analysis of this subject, so as to conduct an in-depth discussion on the value of empowerment education applied to patients after PCI and provide certain reference for future clinical nursing research.

## 2. Aims

Our study intends to evaluate the impact of empowerment education on the quality of life, cognitive level, anxiety and depression level of patients after PCI, and to provide some reference for the choice of clinical nursing mode after PCI.

## 3. Methods

### 3.1. Data sources and search strategies

The following electronic databases were searched by computer: PubMed, Embase, Web of Science, Elsevier Science Direct, The Cochrane Library, CNKI, WanFang Data, and VIP database. The search terms and specific search strategies are shown in Table 1. The search time limitation was from inception to 1 January, 2023. English and Chinese articles were included in this study. A manual search was conducted on the retrieved articles to obtain more relevant research. After importing the article information into EndNote, duplicate articles would be deleted, the titles and abstracts would be screened and cross-checked independently by 2 researchers. If there were any differences, a third party would be invited to solve the problems. The lack of data would be contacted with the original authors, and the full text of the preliminary screened studies would be read to verify whether they meet the inclusion criteria.

**Table 1:** Search strategy used in electronic databases.

Search terms				
Empowerment	**AND**	Percutaneous Coronary Intervention	**AND**	Cardiovascular disease
**OR**		**OR**		**OR**
Empowerment Education		PCI		Heart disease
Patient Empowerment		Stent Intervention		Coronary disease
Patient Participation		Coronary Intervention		Coronary heart disease
Patient Involvement		Interventional operation		CHD
Self-management				Myocardial infarction
Self-care				Acute coronary syndrome
Self-efficacy				ACS
				Angina

### 3.2. Inclusion and exclusion criteria

The research include the published studies on the intervention of empowerment education mode in the patients after PCI, and the literature only includes Chinese and English. The participants were patients over 18 years who underwent PCI due to coronary heart disease (including angina pectoris and myocardial infarction). Regardless of gender, age, race, or nationality. There is no limit to the method, duration, frequency, or content of empowerment education. Outcome indicators included Patients’ quality of life score (SF-36 scale)^[22]^; Self-care ability: Exercise of Self-Care Agency Scale (ESCA)^[23]^; Anxiety and depression status: Self-Rating Anxiety Scale (SAS), Self-Rating Depression Scale, Hospital Anxiety and Depression Scale, Connor-Davidson resilience scale^[24–26]^; CHD cognition level: Disease cognition level questionnaire compiled by American Heart Association Guidelines for PCI,^[27]^ The perceived knowledge scale for CHD^[28]^; medication compliance and postoperative complications. RCTs are preferred in order to obtain sufficient evidence for comparison, but quasi-experimental studies (QES) and Cohort studies are also considered due to the limited number of RCTs. Studies whose data’s were not available, important outcome measures were not adequately stated, and all reviews, protocols, and bibliometric analyses were excluded.

### 3.3. Data extraction and quality assessment

Two researchers screened the literature, extracted the basic information and cross-checked. The differences were discussed and negotiated by the third person. Obviously irrelevant articles were excluded by title during the screening process, and then the abstract and the full text were read to determine whether the articles could be included. If necessary, the authors of the original articles were contacted by email or phone to obtain information about key articles. Two evaluators assessed the bias risk of included studies using The Quality Assessment Tool recommended in the Cochrane handbook updated guidance.^[29]^ Newcastle-Ottawa Quality Assessment Scale^[30]^ was used to evaluate the quality of 2 cohort studies. The JBI Critical Appraisal Checklist^[31]^ was used to assess the quality of QES.

### 3.4. Data synthesis

RevMan5.4 software and R software were used for statistical analysis. Mean difference (MD) or standard MD (SMD) was used as effect analysis statistic for continuous variables, and relative risk was used as effect analysis statistic for dichotomous data with 95% confidence intervals. When homogeneity was found among the included studies (*P* > .10, or *I*^2^ < 50%), they were analyzed by using the fixed effects model. When there is heterogeneity, if *P* < .10 and *I*^2^ > 75%, sensitivity analysis or subgroup analysis should be used to investigate the source of heterogeneity. After excluding the influence of obvious clinical heterogeneity on the study, random effects model was used for meta-analysis. For the outcome indicators with significant heterogeneity, only descriptive analysis was performed if subgroup analysis and sensitivity analysis could not be performed.

### 3.5. Ethical statement

This paper is a systematic review study, which aims to summarize the results of previous studies, so there is no need for consent from patients or the approval of the ethics review committee.

## 4. Results

### 4.1. Literature screening process and results

**Figure 1.**
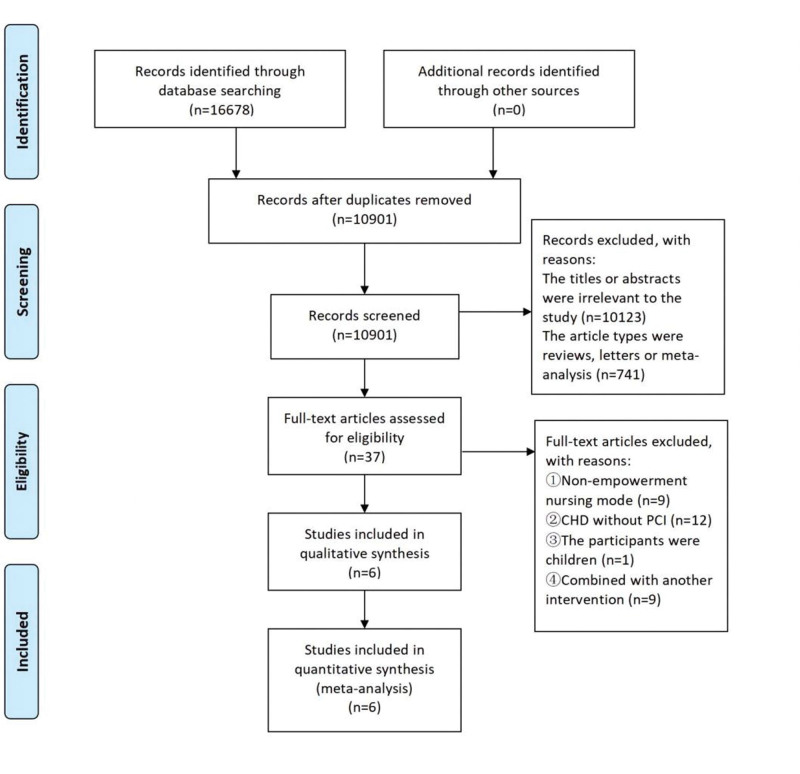
Flow diagram of search and selection of studies. CHD = coronary heart disease, PCI = percutaneous coronary intervention.

A total of 2227 articles were identified by searching the database, 37 articles were obtained after removing duplicates and reading the topic and abstract. Finally, 6 articles with a total of 641 patients were included after the full text reading, including 3 RCTs, 2 cohort studies and 1 QES. 5 English and 1 Chinese articles were included in our study. The literature screening process is shown in Figure 1. Taking PubMed as an example, the specific retrieval process and results are shown in Supplemental Digital Content (see Appendix 1, http://links.lww.com/MD/J109, Supplemental Digital Content, which shows a complete search in PubMed).

### 4.2. Characteristics of the studies and quality assessment

The characteristics of the included studies are shown in Table 2. The Cochrane Collaboration Network RCT bias risk assessment tool^[29]^ was used to assess the bias risk of 3 RCTs. All 3 RCTs explicitly mentioned random measures, but did not mention the implementation of allocation concealment. One RCT mentioned that blinding was not implemented in order to facilitate data collection, while the other two did not. Due to the characteristics of the authorized project, it is impossible to conceal the identity of the researcher from the participants, so we believe that the blinding method for the evaluation of the 3 research results is a high-risk project. The results are shown in Table 3. Newcastle-Ottawa Quality Assessment Scale^[30]^ was applied to evaluate the literature quality of 2 cohort studies, and the results were shown in Table 4. The JBI Critical Appraisal Checklist^[31]^ was used to assess the quality of QES, and the results are shown in Table 5.

**Table 2:** The characteristics of the included studies.

Author, year, country	Design	Participants	Mean age (experimental group/control group)	Experimental group interventions	Control group interventions	Indicator
Sun et al (2022), China	RCT	108 (59)	EG: 61.27 (8.70); CG: 60.91 (8.03)	Empowerment education model	Four face-to-face health education sessions incubated for about 30 min each time	① Exercise of Self-Care Agency Scale (ESCA) ② Self-Rating Anxiety Scale (SAS) ③ Self-Rating Depression Scale (SDS) ④ Medication adherence
Zhu et al (2021), China	Cohort study	148 (80)	RG: 62.73 (3.02); CG: 63.67 (3.15)	Orem’s self-care education	Routine nursing model	① Postoperative complications ② Time of door to balloon (D-to-B) and getting out of bed and discharging ③ The cognition levels: Disease cognition level questionnaire compiled by American Heart Association (AHA) Guidelines for Percutaneous Coronary Intervention ④ Exercise of Self-Care Agency Scale (ESCA) ⑤ Self-Rating Anxiety Scale (SAS) ⑥ Self-Rating Depression Scale (SDS)
Wang et al (2021), China	RCT	85 (69)	EG: 60.50 (8.90); CG: 60.86 (8.85)	Empowerment-based intervention was developed based on the Empowerment Process Model	Routine nursing model. Its main strategy was general health education	① The cognition levels. Perceived knowledge scale for coronary heart disease (PKS-CHD) ② Connor-Davidson resilience scale CD-RISC
Jiang et al (2020), China	QES	102 (86)	EG: 54.75 (10.29); CG: 55.18 (8.20)	Nurse-led individualized self-management program (NISMP). NISMP was a well-structured 12-mo program that consisted of 6 group-based education sessions	Usual care included the usual discharge plan provided by a nurse, and regular medical appointments with a cardiologist every 3 mo at the outpatient clinic	① Health-Promoting Lifestyle Profile II (HPLPII) ② Risk Factors Assessment Form (RFAF) ③ Low-Density Lipoprotein (LDL) ④ Blood pressure (BP) ⑤ Blood glucose (BG) ⑥ Body mass index (BMI) ⑦ The SF-36 quality of life scale
Takematsu et al (2015), Japan	Cohort study	138 (112)	RG: 72.3 (7.6); CG: 67.2 (9.1)	Self-care education	Routine nursing model	① The SF-36 quality of life scale
Furuya et al (2015), Brazil	RCT	60 (34)	EG: 63.3 (12.4); CG: 60.6 (8.7)	Self-care educational programme with telephone follow-up	Usual care. Routine instructions given by the hospital	① The SF-36 quality of life scale ② Hospital Anxiety and Depression Scale (HADS) ③ Medication adherence: Medida de Adesao aos Tratamentos (MAT;Treatment Adherence Measure)

CG = Control group, EG = Experimental group, QES = Quasi-Experimental Study, RCT = randomized controlled trial.

**Table 3:** Bias risks of randomized controlled trials.

Study	Random sequence generation (selection bias)	Allocation concealment (selection bias)	Blinding of participants and personnel (performance bias)	Blinding of outcome assessment (detection bias)	Incomplete outcome data (attrition bias)	Selective reporting (reporting bias)	Other bias
Sun et al (2022)	Low risk	Unclear	High risk	High risk	Low risk	Low risk	Low risk
Wang et al (2021)	Low risk	Unclear	High risk	High risk	Low risk	Low risk	Low risk
Furuya et al (2015)	Low risk	Unclear	High risk	High risk	Low risk	Low risk	Low risk

**Table 4:** Bias risks of cohort studies.

Study	Selection				Comparability	Outcome			Total
	Representativeness of the exposed cohort	Selection of the non-exposed cohort	Ascertainment of exposure	Demonstration that outcomes were not present at the start of study	Comparability of cohorts on the basis of the design or analysis	Assessment of outcome	Adequate follow-up duration	Adequacy of follow-up of cohorts	Score
Zhu et al (2021)	1	1	1	1	1	1	1	1	8
Takematsu et al (2015)	1	1	1	1	1	1	1	0	7

**Table 5:** Bias risks of quasi-experimental studies.

Study	Q1	Q2	Q3	Q4	Q5	Q6	Q7	Q8	Q9
Jiang et al (2020)	YES	YES	NO	YES	YES	NO	YES	YES	YES
Q1: Is it clear in the study what is the “cause” and what is the “effect?” Q2: Were the participants included in any comparisons similar? Q3: Were the participants included in any comparisons receiving similar treatment/care, other than the exposure or intervention of interest? Q4: Was there a control group? Q5: Were there multiple measurements of the outcome both pre and post the intervention/exposure? Q6: Was follow up complete and if not, were differences between groups in terms of their follow up adequately described and analyzed? Q7: Were the outcomes of participants included in any comparisons measured in the same way? Q8: Were outcomes measured in a reliable way? Q9: Was appropriate statistical analysis used?									

### 4.3. Meta-analysis for outcomes measures

#### 4.3.1. Quality of life.

A total of 3 studies assessed patients’ quality of life, all of them using SF-36 assessment scale. Since the scale was composed of 8 sub-items, patients’ quality of life was evaluated from 8 aspects: general health (GH), physical function, role physical (RP), role emotional, social function (SF), bodily pain, vitality, and mental health (MH). Therefore, our study conducted meta-analysis on the subitems of SF-36 score, and the results of each study were shown in Table 6.

**Table 6:** Meta-analysis on the subitems of SF-36.

The content of SF-36	Heterogeneity test		Effect model	Meta-analysis (95% CI)	
	*P*	*I* ^2^		MD (95% CI)	*P*
GH	.78	0	Fixed	5.83 [3.26, 8.40]	<.00001
PF	.61	0	Fixed	2.43 [0.70, 4.15]	.006
RP	.03	0.72	Random	5.51 [−4.56, 15.58]	.28
RE	.07	0.62	Random	6.68 [−2.35, 15.71]	.15
SF	.01	0.77	Random	6.40 [−1.77, 14.56]	.12
BP	.68	0	Fixed	3.81 [0.92, 6.69]	.01
VT	.16	0.46	Fixed	6.80 [4.93, 8.68]	<.00001
MH	.22	0.35	Fixed	5.82 [3.95, 7.68]	<.00001

BP = bodily pain, CI = confidence interval, GH = general health, MD = mean difference, MH = mental health, PF = physical function, RE = role emotional, RP = role physical, SF = social function, VT = vitality.

#### 4.3.2. Self-care ability.

**Figure 2.**
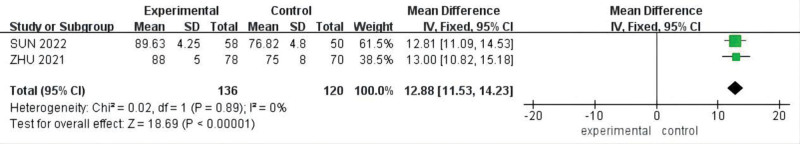
Meta-analysis of the ESCA. ESCA = Exercise of Self-Care Agency Scale.

A total of 2 studies reported patients’ self-care ability, both of which used ESCA score as outcome index. Heterogeneity test: *P* = .89, *I*^2^ = 0%. Meta-analysis using fixed effect model showed that the ESCA score of the experimental group was higher than that of the control group, with statistically significant difference (MD = 12.88, confidence intervals [11.53, 14.23], *P* < .00001). The results were shown in Figure 2.

#### 4.3.3. Cognitive level of CHD.

**Figure 3.**
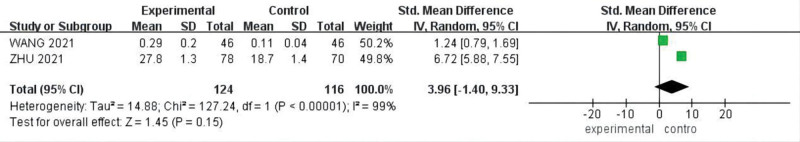
Meta-analysis of the cognitive level of CHD. CHD = coronary heart disease.

There were 2 studies evaluating the cognitive level of patients with coronary heart disease. Zhu et al (2021) according to the Disease cognition level questionnaire compiled by American Heart Association Guidelines for PCI. Wang et al (2021) used perceived knowledge scale-CHD to evaluate the cognitive level of patients. The above 2 studies were combined for analysis. The statistics of effect analysis was conducted by SMD, the heterogeneity test *I*^2^ = 99%, *P* < .00001, so the random effects model was used. The results showed that empowering education could increase the knowledge of coronary heart disease in patients after PCI (SMD = 3.96 [−1.40, 9.33]). But the difference was not statistically significant (*P* = .15). The results were shown in Figure 3.

#### 4.3.4. Anxiety-depressive state.

There were 2 studies using Self-Rating Anxiety Scale and Self-Rating Depression Scale scores to assess anxiety and depression. One study used the Hospital Anxiety and Depression Scale to assess anxiety and depression. Since the scores of the above 2 scales are positively correlated with the degree of anxiety and depression, the above 3 studies were combined for analysis, and SMD was used for effect analysis statistics. The meta-analysis results showed that empowerment education could significantly reduce the anxiety scores of patients after PCI, but did not significantly improve the depression scores. The results are shown in Table 7. Another study assessed the mental resilience of patients using the Connor-Davidson resilience scale, which is designed to measure the individual’s ability to resist stressful life events in life. Compared to the score at baseline (T1), only the intervention group had significantly increased score at completion of the intervention (T2) (*t* = 6.184; *P* < .001). Significant difference (*t* = 3.235; *P* = .002) was evident in the score changes (T2–T1) between the control group (mean = 1.88; SD = 12.12) and the intervention group (mean = 9.76; SD = 10.23).

**Table 7:** Meta-analysis of the Anxiety-depressive state.

Mental state	Heterogeneity test		Effect model	Meta-analysis (95% CI)	
	*P*	*I* ^2^		SMD (95% CI)	P
Anxiety	.13	52%	Random	−1.65 [−2.04, −1.27]	<.00001
Depression	<.00001	97%	Random	−1.35 [−2.84, 0.14]	.08

CI = confidence interval, SMD = standard mean difference.

### 4.4. Qualitative synthesis of other outcomes.

#### 4.4.1. Medication adherence.

A total of 2 studies reported medication adherence, but Furuya et al (2015) only mentioned that “*In both groups, 94 % of the participants reported medication adherence at baseline and 6-month follow-up*,” did not provide detailed data on compliance comparisons between the 2 groups. Therefore, our study only reports the research results of Sun et al (2022). In Sun’s research, the Morisky medication compliance questionnaire prepared by Professor Morisky was used to evaluate the medication adherence of patients. The 2-point scoring system was adopted, and the score of “yes/no” for each item was “1/0,” with the total score ranging from 0 to 8. The higher the score, the better the patient’s medication compliance. At discharge, the difference of scores between the 2 groups was not statistically significant (*P* > .05). Three months after discharge, the medication adherence of patients in the empowerment education group after PCI was higher than that in the control group, the difference was statistically significant (*F* = 7.266, *P* < .001).

#### 4.4.2. Postoperative complication.

Zhu et al (2021) reported the complications after PCI, including 2 cases in the empowerment group with puncture site bleeding and 8 cases in the control group, 2 cases in the empowerment group with postoperative hypotension and 7 cases in the control group, 3 cases in the empowerment group with arrhythmia and 8 cases in the control group, 1 case in the empowerment group with lower limb deep vein thrombosis and 5 cases in the control group. 2 cases in the empowerment group with urinary retention and 10 cases in the control group. Based on the above results, the incidence of complications in the experimental group was 12.81%, significantly lower than that in the control group 54.29% (*P* = .05).

### 4.5. Publication bias and sensitivity analysis

**Figure 4.**
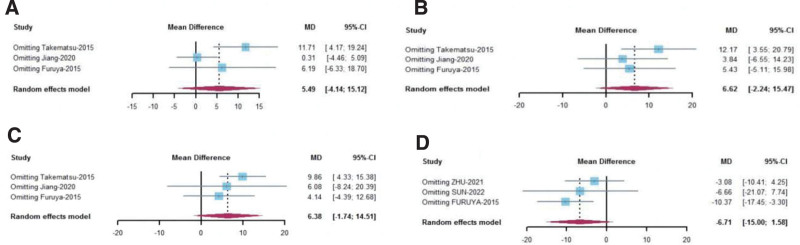
The results of sensitivity analysis.

For studies with heterogeneity test *I*^2^ > 75%, We used R software for publication bias test and sensitivity analysis. The outcome measures included RP, role emotional, SF, and depression score. The results showed that the publication bias test *P* value of the above 4 outcome indicators were all greater than .005, indicating no publication bias. The results were shown in Table 8. Sensitivity analysis showed that the heterogeneity of RP was derived from the study of Furuya et al (2015), but repeated attempts failed to contact the author. Sensitivity analysis of other indicators indicates that the results are stable, as shown in the Figure 4.

**Table 8:** The results of publication bias test.

Outcomes	*t*	df	*P* value
RP	0.42	1	.7480
RE	2.23	1	.2684
SF	1.8	1	.3227
Depression	0.43	1	.7428

RE = role emotional, RP = role physical, SF = social function.

## 5. Discussion

Our study is the first systematic review and meta-analysis of empowerment education to guide rehabilitation after PCI, including 5 English studies and 1 Chinese study. Empowerment education is a process that enables patients to take control of their lives by stimulating internal behavior change.^[32]^ Because patients have more autonomy in the treatment, nursing and rehabilitation process, there will be a greater degree of influence on their health.^[33,34]^ Researchers have summarized empowerment education into 5 steps, including clarifying problems, expressing feelings, setting goals, making plans and evaluating effect.^[35]^ Numerous clinical studies have confirmed that the patients after PCI can benefit greatly from lifestyle changes, rational rehabilitation training and risk reduction behaviors.^[36–39]^ Nurses can play a key role in education and care, supporting and facilitating patient participation in these risk reduction activities.^[40]^ Empowerment education is an innovation and upgrade of the previous conventional nursing education. Through empowerment, patients after PCI are better in physically and psychologically than conventional nursing education.^[41]^

The SF-36 is a very popular tool for assessing health-related quality of life.^[42]^ A PubMed search with the keyword “SF-36 health survey” showed 14,797 records. In a prospective study followed for 8 years at a Danish community hospital, the SF-36 scale was associated with a reduced risk of CHD, with individuals with the highest SF-36 activity quartile having a 38% reduced risk of CHD (HR: 0.62 [0.42; 0.93]) compared to individuals with the lowest SF-36 activity quartile.^[43]^ Therefore, combined with the results of the current study, there seems to be compelling evidence that satisfaction with life, specifically SF-36 scale scores, are important in the assessment of CHD.^[44]^ This study showed that in 8 items of the SF-36 scale, empowerment education significantly improved GH, vitality, MH, physical function and bodily pain in patients after PCI, with statistical significance, while the improvement of the other 3 items was not statistically significant. Bodily Pain is one of outcomes in the SF-36 scale, our study has showed that empowerment education could improve patients’ postoperative pain to some extent.^[45]^ A recent another study showed that recurrent chest pain was a common cause of readmission in patients discharged after PCI.^[46]^ Some of these readiness’s are due to progression or complications of atherosclerotic disease (restenosis and stent thrombosis) after successful PCI, however, some are avoidable.^[47]^ A systematic review of 24 studies found that 27% of readmissions could have been avoided through patient education, medication, post-discharge follow-up, and telephone follow-up, which reminds us of the importance of nursing education for discharged patients.^[48]^ Empowerment education aims to strengthen the prevention concept through more advanced nursing education, which proposed by 2020 European Society of Cardiology guidelines^[49]^ for cardiac re habilitation.

In 2020, a Norwegian study of continuity of care for discharged patients after PCI found that as patients after PCI move between hospital and community, the potential for discontinuity arises, and the healthcare system needs to take more responsibility to educate and counsel patients.^[50]^ An another survey study of 238 patients after PCI found that patients’ health literacy and self-care ability were important factors affecting patients’ quality of life after PCI.^[51]^ Further study found that patients with high health literacy were more likely to improve related quality of life through adequate knowledge of drugs, but patients with poor health literacy were more likely to make conflicting clinical decisions.^[52]^ From the above studies, it can be found that the cognitive level and self-care ability of discharge patients are helpful for follow-up rehabilitation, and scientific and effective nursing education is an important guarantee for this. So our study conducted a meta-analysis on the self-care ability and cognitive level of coronary heart disease of patients after PCI, and the results showed that empowerment education could improve patients’ self-care ability, but there was no significant difference in improving the cognitive level of CHD. Because only 2 studies were included, it is considered that our result may be related to the lack of literature inclusion and the high heterogeneity of the studies. In particular, for the cognitive level of coronary heart disease, the heterogeneity test *I*^2^ = 99% due to the differences in the questionnaire survey directions adopted in the 2 studies. Therefore, more large-sample and high-quality studies are needed for this index in the future.

Anxiety and depression is a common psychological state. Generally, CHD patients are more prone to mental disorders, because they usually endure unpleasant symptoms such as angina without warning, and need to take several drugs throughout their life.^[52]^ After PCI, due to the worry of surgery and the lack of cognition, it is often easy to lead to anxiety, expression or other negative emotions.^[53]^ Specifically, 20% to 30% of patients with heart disease were diagnosed with anxiety or depression, and the proportion of patients with anxiety and depression rose to 15% to 43% in the 12 months following an acute heart attack.^[54]^ Our study conducted a meta-analysis of the 3 included studies, and the results showed that empowerment education had a statistically significant effect on the anxiety state of patients after PCI, but had no statistically significant effect on the change of depression state. A recent study on anxiety after PCI found that although patients’ anxiety can be relieved after PCI to a certain extent, smokers, young people and those with low education level are still easy to maintain a high level of anxiety.^[55,56]^ Therefore, it is more significant to carry out education, popularize knowledge about CHD, quit smoking and implement exercise guidance for these anxious groups in the future.^[57]^

Because of the broad meaning of empowerment education, different studies have slightly different interventions. We noted that Jiang and Furuya’s study, which combined a 12-month telephone follow-up after empowerment, showed benefits in improving postoperative SF-36 scores and anxiety. However, limited by the number of studies available, we were unable to compare the benefits of telephone follow-up with those of empowerment education. Considering that telephone follow-up may bring pressure on medical and nursing resources, we suggest that a reasonable empowerment education model should be formulated according to the specific situation of the number of patients and medical resources in medical institution, so as to achieve a balance between the benefits of patients and the medical burden. For high-risk groups after PCI, such as contrast-induced nephropathy,^[58]^ should focus on education.

## 6. Limitations

There are also some limitations in our study: although the overall quality of the included literature is high, the implementation of the allocation concealment and blinding is difficult to some extent due to the restriction of intervention measures, which may lead to bias in the implementation measurement. There were differences in the evaluation scales of some outcome indicators, leading to certain clinical heterogeneity. Some outcome indicators are included too little studies, resulting in the low stability of the conclusion.

## 7. Conclusion

Applying the concept of empowerment to postoperative education of PCI patients can improve patients’ quality of life. The GH condition, physiological function, physical pain, vital vitality and MH of the patients were improved by empowerment education. Our study shows that postoperative empowerment can improve the self-care ability of patients with PCI, which may be achieved by improving the cognitive level of patients with coronary heart disease and enhancing medication compliance. In addition, empowerment education significantly improved the anxiety state of patients after surgery, but did not improve the depression state. Because empowerment education is relatively safe for patients, we suggest that empowerment education can be applied to the postoperative education of patients with PCI, so as to carry out more large-sample, multi-center clinical trials.

## 8. Relevant to clinical practice

Compared with the traditional nursing education mode, empowerment education brings more autonomy to patients on the basis of full and comprehensive rehabilitation theory education. This patient-oriented nursing concept is of great significance in the process of chronic disease rehabilitation. In clinical nursing, patients often have excessive emotional reactions due to lack of knowledge of the disease, or reject some drugs that need to be taken for a long time with benefits due to lack of understanding of medicine knowledge with the process of rehabilitation after PCI. At the same time, because of the rapid development of cardiac rehabilitation technology, it is difficult for clinicians to give patients a full use of their own advantages of the rehabilitation program. Therefore, the review suggests applying the empowerment nursing mode to allow patients to take themselves as the center, fully mobilize their own rehabilitation resources, and better cooperate with clinicians’ treatment plans.

## Author contributions

**Data curation:** Linbin Guo, Tianlin Wang, Xinjue Shan.

**Formal analysis:** Linbin Guo, Wanpeng Gao, Tianlin Wang.

**Investigation:** Linbin Guo.

**Methodology:** Linbin Guo, Wanpeng Gao, Xinjue Shan.

**Resources:** Wanpeng Gao, Tianlin Wang.

**Software:** Linbin Guo, Xinjue Shan.

**Validation:** Linbin Guo, Tianlin Wang.

**Visualization:** Linbin Guo, Tianlin Wang, Xinjue Shan.

**Writing – original draft:** Linbin Guo.

**Writing – review & editing:** Linbin Guo

## Supplementary Material


