# Supplementary material for: Effects of empowerment education on patients after percutaneous coronary intervention: A meta-analysis and systematic review
Source: Medicine (Baltimore). 2023 Jun 9;102(23):e33992. doi: 10.1097/MD.0000000000033992 (PMC10256392; doi:10.1097/MD.0000000000033992)
Supplement: Supplementary file 1 [file medi-102-e33992-s001.pdf]

Appendix 1. A complete search in PubMed.

	PubMed with daily update<br>Searches	Results	Type
1	Empowerment	17336	advanced
2	Empowerment education	7735	advanced
3	Patient Empowerment	70623	advanced
4	Patient Participation	66477	advanced
5	Patient Involvement	155048	advanced
6	Self-management	100806	advanced
7	Self-care	240471	advanced
8	Self-efficacy	81689	advanced
9	1or2or3or4or5or6or7or8	491189	advanced
10	Percutaneous Coronary Intervention	78587	advanced
11	PCI	33693	advanced
12	Stent Intervention	95070	advanced
13	Coronary Intervention	794575	advanced
14	Interventional operation	2875000	advanced
15	10or11or12or13or14	3361035	advanced
16	Cardiovascular disease	2877973	advanced
17	Heart disease	1516937	advanced
18	Coronary disease	360149	advanced
19	Coronary heart disease	360149	advanced
20	CHD	31066	advanced
21	Myocardial infarction	279665	advanced
22	Acute coronary syndrome	41140	advanced
23	ACS	147425	advanced
24	Angina	73988	advanced
25	16or17or18or19or20or21or22or23or24	3186905	advanced
26	9and15and25	15597	advanced
